# Perinatal Outcome of Pregnancies Complicated by Immune Thrombocytopenia

**Published:** 2012-07-30

**Authors:** B Namavar Jahromi, Z Shiravani, L Salarian

**Affiliations:** 1 Perinatology Research Center, Department of Obstetrics and Gynecology, School of Medicine, Shiraz University of Medical Sciences, Shiraz, Iran; 2 Department of Obstetrics and Gynecology, School of Medicine, Shiraz University of Medical Sciences, Shiraz, Iran

**Keywords:** Immune thrombocytopenia, Perinatal, Outcome, Iran

## Abstract

**Background:**

Immune thrombocytopenia (ITP) is an autoimmune disorder that leads to premature destruction of antibody-coated platelets. This study evaluated perinatal outcome and medications used for pregnancies complicated by ITP.

**Methods:**

Medical records of 132 pregnancies belonged to 125 parturients with ITP who delivered between March 2001 and January 2011 were reviewed. Cases were included if diagnosed before pregnancy or if their platelet counts (PCs) were less than 80,000/µL during pregnancy without any other cause. Maternal and fetal outcomes were compared.

**Results:**

Fifty six mothers (42.1%) had PC<50,000, 18 women (13.5%) developed preeclampsia and 15 (11.3%) were diabetics. Corticosteroid was used for120 cases (90.9%) and intravenous immunoglobulin for 14 women (10.5%). PCs of 114 neonates were available in the charts and 84 (83.2%) had PC>150,000/µL. Three neonates (2.3%) had PC<50 000, 31 neonates (23.3%) had preterm births and 32 (24.1%) needed NICU admissions. Fifty seven cases of ITP (43.2%) were diagnosed before pregnancy and 75 (56.8%) were diagnosed during pregnancy. There were 2 intrauterine fetal deaths and higher NICU admissions, 20 (34.48%) versus 12 (16%) in the first group (p=0.01).

**Conclusion:**

Perinatal outcome of pregnancies with ITP is generally good. However neonates born from parturients with chronic ITP needed more NICU admissions.

## Introduction

Immune thrombocytopenia (ITP) is an autoimmune disorder that leads to premature destruction of anti-body-coated platelets. For the diagnosis of ITP there is no approved confirmatory laboratory test at present. Diagnosis is made by demonstrating an isolated thrombocytopenia without an obvious cause. Antiplatelet antibodies may cross the placenta and cause bleeding complications in the neonate.[1][2] Pregnancy with ITP is sometimes difficult to manage due to bleeding potentials for the mother and her fetus during peripartum periods. Thrombocytopenia occurs in 7-10% of unselected pregnancies. However, ITP accounts for only 3% of these cases compared to gestational thrombocytopenia and hypertensive disorders of pregnancy that account for 74% and 21% of the cases, respectively.[3][4] This project was designed to retrospectively study the outcome of pregnant women with ITP and their neonates during an almost 10-year period.

## Materials and Methods

This study was performed retrospectively from March 2001 to January 2011 in Hafez and Hazrate Zeinab hospitals affiliated to Shiraz University of Medical Sciences, Shiraz, Southern Iran. Thrombocytopenia was defined as platelet count (PC) of less than 150,000/µL. Patients were included if they were known cases of chronic ITP prior to pregnancy or were diagnosed during pregnancy with PCs of less than 80,000/µL for which other causes were excluded.[5][6][7] Other causes of thrombocytopenia include sepsis, HEELP (hemolysis, elevated liver enzymes and low platelet) syndrome, disseminated intravascular coagulation (DIC), drug induced thrombocytopenia, and thrombocytopenia associated with autoimmune diseases such as systemic lupus erythematosis, thrombotic thrombocytopenia, hemolytic uremic syndrome and hereditary forms of thrombocytopenia.[8]

Incidental or gestational thrombocytopenia was diagnosed when thrombocytopenia was first diagnosed during pregnancy in an otherwise asymptomatic patient with platelet counts of more than 80,000/µL.[5][6] These cases were excluded from this study. According to the PCs, the mothers and their neonates were classified to have mild (100,000≤PC<150000/µL) moderate (50,000≤PC<100 000/µL) and severe (PC<50,000/µL) thrombocytopenia.

A woman was considered to have preeclampsia if her blood pressure was ≥140/90 with urine protein of more than 300 mg in 24 hours or more than 1+ dipstick.[9] Women with overt or gestational diabetes who were diagnosed according to ACOG criteria [10] were analyzed as diabetic group. If delivery occurred before 259 days of gestational age, it was considered as a preterm birth (PTB).[11] Gestational ages were calculated from the first day of the last menstrual period and were confirmed by first trimester ultrasound scans.

The extracted data included maternal PC at delivery, history of ITP prior to pregnancy, prior splenectomy, therapeutic modalities, mode of delivery and complications, neonatal PC at birth, gestational age at delivery, neonatal birth weight and NICU admission.

This project was approved by the Ethics Committee of Shiraz University of Medical Sciences. Statistical analyses were performed by SPSS software (Version 16, Chicago, IL, USA). Evaluation of correlation between maternal and neonatal PCs was performed by Pearson correlation test. Evaluation of correlation between previous splenectomy in the mother and neonatal PC was performed by independent sample t-test. Chi-Square test was used for comparison of ratios that were presented as number (%) and for comparison of means, independent sample t test was used. Statistical significance was set at p<0.05.

## Results

One hundreds and thirty two pregnancies were included from125 women with ITP, 7 women had two deliveries and one was twin. Four singleton women did not deliver in our centers, so 129 neonates remained for the final analysis. Fifty seven pregnancies from 132 (43.2%) were known cases of ITP before pregnancy and 75 (56.8%) were diagnosed during pregnancy. The mean gestational age at delivery was 266.9±14.7 days (range: 206-294 days). Eighteen women (13.5%) were considered to have preeclampsia and 15 (11.3%) were diabetic. Several maternal and neonatal data were presented in Table 1.

**Table 1:** Several maternal and neonatal features of pregnancies complicated by ITP at the time of delivery.

**Variable**	**mean±SD**	**Range**
Maternal age (years)	26.5± 4.8	16-40
Maternal platelet count at delivery (/µL)	62,260±38,642	3,000-200,000
Gestational age at delivery(days)	266.9±14.7	206-294
Neonatal birth weight (grams)	29189±588.36	1150-3900
Neonatal platelet count (/µL)	146,040±60,995	36,000-314,000

There were 9 women (6.8%) with PC>150,000/µL. Eight mothers (6%) had mild, 59 (44.4%) had moderate and 56 (42.1%) had severe thrombocytopenia. The platelet counts of 114 neonates were available in the charts. There were 84 neonates (83.2%) with PC≥150,000/µL. Fourteen neonates (10.5%) had mild, 13 (9.8%) moderate and 3 (2.3%) severe thrombocytopenia, respectively. The 3 neonates with PC<50,000/µL were born from the mothers with ITP prior to pregnancy. No neonates had severe bleeding complications and no intracranial hemorrhage occurred.

There was no significant correlation between maternal platelet count and neonatal platelet count (p=0.19, r=0.15). Also, there was no statistically significant correlation between previous splenectomy and neonatal platelet counts at birth (p=0.64).

Thirty-one (23.3%) neonates had PTB and 32 (24.1%) infants had NICU admissions. Eight neonates (6%) had Apgar Scores below 7 at 5 minutes. There were 2 fetuses that had intrauterine deaths on admission time and were included among the cases with Apgar scores below 7. Seventy four pregnancies (55.6%) were terminated by cesarean and 54 (40.6%) had vaginal deliveries. Indications for abdominal deliveries were fetal distress (n=32, 43.2%), previous cesareans (n=27, 36.5%), abnormal presentations (n=6, 8.1%), active phase arrest (n=4, 5.4%), twin (n=1, 1.4%), prolonged rupture of membranes and not responsive to induction of labor (n=4, 5.4%).

Medications that were used for the study group during the admission time were corticosteroids including prednisolone (n=88, 66.2%), dexamethasone (n=78, 58.6%) and hydrocortisone (n=39, 29.3%). Fourteen women (10.5%) used intravenous immunoglobulin (IVIg), 38 women (28.6%) used at least one kind of medication, 66 (49.6%) used two kinds, 15 (11.3%) used three kinds and for one case (0.8%), 4 types of medications were used simultaneously. There were only 11 women (8.3%) who did not use any kind of medications. Four of them were diagnosed before pregnancy and 7 cases diagnosed during pregnancy. There were 8 (6%) women known cases of ITP who had splenectomy prior to their pregnancies. Sixty three women (47.4%) received platelet transfusion in their hospital course.

There were 57 (43.2%) cases who were known cases of ITP before pregnancy and 75 (56.8%) were diagnosed during pregnancy. These two groups were compared. The data was presented in Table 2. The two groups had no statistically significant difference except for NICU admission rate which was higher for the neonates of the women who were known cases of ITP prior to pregnancy (p=0.01).

**Table 2:** Comparison of maternal and neonatal characteristics of pregnancies complicated by ITP between the cases that were diagnosed before pregnancy with the cases diagnosed during pregnancy.

**Variable**	**Diagnosed before pregnancy (n=58)**	**Diagnosed during pregnancy (n=75)**	***P* value^c^**
Maternal age (year)	26.08±5.09	26.8±4.64	0.416
Maternal PCs (/µL)	64,035±44,941	60,920±33,327	0.648
Gestational age at delivery (days)	264±17.3	267±14.3	0.204
Preterm birth	16 (27.6%)	15 (20%)	0.416
Diabetes	9 (15.5%)	6 (8%)	0.165
Preeclampsia	7 (12.1%)	11 (14.7%)	0.695
Cesarean delivery	35 (60.3%)	39 (52%)	0.348
5 minutes apgar score<7	3 (5.2%)	5 (6.7%)	0.715
Neonatal birth weight (grams)	2915.7±678	2926.6±526.3	0.924
Neonatal PC^a^ (/µL)	135880±58899	153860±62605	0.924
Neonatal PC^a^<50,000/µL	3 (2.3%)	0 (0.0%)	0.122
Maternal PC^a^<50,000/µL	25 (43.1%)	31 (41.3%)	0.447
NICU^b^ admission	20 (34.48%)	12 (16%)	0.017

^a^ PC: Platelet Count

^b^ NICU: Neonatal Intensive Care Unit

^c^ Values are presented as mean±SD or number (%)

There were two women admitted with intrauterine dead fetuses at 224 and 247 days of pregnancy. They were both known cases of ITP prior to pregnancy. In this study, there were 5 women with bleeding complications who needed blood transfusions. The first was a 28 years old woman with PC=20,000/µL who needed emergency cesarean for fetal distress and developed hematoma at the site of spinal needle. The second case had normal delivery with PC=41,000/µL and developed postpartum uterine bleeding and needed transfusion. The other 3 cases had cesarean deliveries for obstetric indications with PCs of 15,000/µL, 45,000/µL and 70,000/µL and developed hemorrhage during operation. The parturient with platelet count of 70,000/µL needed reoperation for drainage of massive hematomas. There were no maternal mortalities among this study population.

## Discussion

ITP is estimated to occur in 1 in 1000 to 1 in 10 000 pregnant women and is responsible for almost 3% of all cases of thrombocytopenia in pregnancy and considered as a rare condition.[6][12] Careful management of ITP during pregnancy may become life saving for the mother and her offspring. Diagnosis of ITP is a diagnosis of exclusion and should be performed clinically. According to practice guidelines platelets, rarely decrease to less than 80,000/µL in gestational thrombocytopenia.[5][6] In this study, if thrombocytopenia presented for the first time during pregnancy, we included only the cases with PCs less than 80,000/µL. However the cases that were diagnosed prior to pregnancy were all included. We retrospectively reviewed the admission charts of the patients in an almost 10-year period and 132 pregnancies belonged to 125 mothers were included. Fifty-eight cases were diagnosed before pregnancy and 75 were diagnosed during pregnancy.

The maternal ages ranged between 16-40 years (mean 26.49±4.8 years) and maternal platelet counts were between 3,000-200,000/µL (mean 62,850±39,081/µL). However, 115 women (86.6%) were classified to have moderate to severe thrombocytopenia with platelet counts of below 100,000/µL and 56 of them (42.1%) had PC< 50,000/µL. On the contrast, among the neonates with the mean platelet count of 146,040±60,995/µL, one hundred and thirteen (84.9%) had platelet counts of more than 100,000/µL. Thirteen neonates (9.8%) had platelet counts between 50,000-100,000/µL and only 3 (2.3%) had platelet counts below 50,000/µL. None of these neonates showed severe bleeding complications and there was no case of intracranial hemorrhage. However, higher rates had been reported for severe neonatal thrombocytopenia in previous studies.[13] Neonatal PC may drop during few days after birth and the difference may be caused by measuring PCs at different durations from delivery. This low rate of fetal severe thrombocytopenia emphasizes that performing invasive procedures before delivery for the measurement of fetal PCs may not be justified. Analysis of our data showed no correlation between maternal and neonatal platelet counts.

Although the mean gestational age at delivery was 266.4±15.6 days in this study, 31 neonates (23.3%) had PTBs and 32 neonates had NICU admissions mostly due to prematurity and its complications. The incidence of PTB in most developed countries is about 5-10%.[14] However, higher PTB rates have been reported for some populations,[15] but it is estimated to be about 5% in the general population in our centers.[16] The calculated PTB rate of 23.3% for the pregnancies complicated by ITP in this study was four times higher.

Among this study group, there were 18 women (13.6%) who developed preeclampsia. Regarding the fact that hypertensive disorders complicate 5-10% of pregnancies,[8] the calculated rate of 13.6% for the parturients with ITP is more than general population. In this study, there were 15 women (11.4%) diagnosed to have either impaired glucose tolerance test or overt diabetes. Compared to the rate of diabetes estimated to be about 4.2%,[17] this study showed a higher tendency for impaired glucose metabolism in this population. Higher rates of preeclampsia and diabetes in this group may be consequences of associated vascular problems or as a complication of corticosteroid therapy.

Antiplatelet antibodies may cross the placenta and cause thrombocytopenia and bleeding complications in the neonate. Obstetricians are always worried about birth trauma and especially ICH in a thrombocytopenic neonate. From a historical point of view, it was suggested to perform umbilical blood sampling near term [18] and cesarean delivery was considered to be indicated for the thrombocytopenic fetuses or for the mothers who have positive circulating platelet antibody test.[19] However evidence showed that invasive tests have little effect in preventing neonatal complications [20][21] and it is now a general agreement that vaginal delivery is preferred for the parturients with ITP unless an obstetric indication occurs.[22][23] In this study, 55 women (41.4%) had normal vaginal deliveries and 74 cases (55.6%) delivered abdominally. The most frequent indication for cesareans was fetal distress that occurred for 32 cases (43.2%).

The mean neonatal birth weight was 29189±588.36 grams. Five minutes apgar scores below 7 was seen only in 8 neonates (6%) including 2 dead fetuses. These two fetuses that were dead on admission in addition to the three neonates that had platelet counts less than 50,000/µL were all born from the mothers with chronic ITP. So, we sub-classified all of the women to two groups (diagnosed before pregnancy and diagnosed during pregnancy) and compared the outcomes between them. We noticed that maternal age, maternal platelet counts, gestational age at delivery, 5 minutes apgar scores<7, rate of abdominal deliveries, mean neonatal birth weights and the mean neonatal platelet counts were not statistically different between the two groups. However, NICU admissions were significantly higher in the group that were diagnosed before pregnancy, 20 (34.48%) compared to 12 (16%) (p=0.01). Suggesting that fetus in a chronic case may probably be more compromised and fetal well being evaluations are more strongly recommended for this group during pregnancy.

Eleven women (8.3%) did not receive any medication. However, the most frequently used medication were corticosteroids (n=121, 91.7%). Fourteen women (10.5%) who did not respond to corticosteroids to rise their platelet counts received IVIG during pregnancy. Sixty three (47.4%) had platelet transfusions at the time of normal delivery or cesarean. Some patients in this study were over-treated compared to the clinical guidelines,[5][6][24] which reflects the fear of the medical team from low PCs and its probable complications during delivery. According to the clinical practice guidelines PC>20,000 during pregnancy and PC>50 000 near term and PC>80,000 for epidural anesthesia is targeted. However, counts may drop unpredictably and major bleeding has been reported rarely even with platelet counts that seem appropriate.[25] Furthermore obstetric hemorrhage may happen due to placental abruption or previa, uterine atonia or uncontrollable genital lacerations even in a normal coagulating condition. In this study, there were two cases that had platelet counts above the targeted values but developed bleeding complications. The first case developed severe vaginal bleeding after normal delivery with PC=41,000/µL and the second case had severe hemorrhage in abdominal incision site with PC=70,000/µL. However, the final maternal and neonatal outcome was good. We believe that hypercoaguable state of pregnancy may somehow protect the mothers with ITP to develop fewer bleeding complications compared to non-pregnant conditions.

This study had several limitations. The first is its retrospective nature and absence of some data in the charts. The second is the possibility of misdiagnoses. If thrombocytopenia was diagnosed for the first time during pregnancy, the case was included only if PC was less than 80,000/µL. There is the possibility that some real cases of mild ITP had not been included or some cases of gestational thrombocytopenia had been included in error. The third limitation is that we were not able to follow the neonatal PCs after delivery and other late neonatal outcomes were not studied.

Maternal and neonatal outcomes of pregnancies complicated by ITP are generally good. All of the cases should be carefully managed by a team of hematologist, neonatologist, anesthesiologist and obstetrician for the best results. However, fetal well being evaluations are more strongly recommended for the chronic cases.
